# Liberality of Parke, Davis & Co.

**Published:** 1887-04

**Authors:** 


LIBERALITY OF PARKE, DAVIS & CO.—Among the prizes conferred at the late commencement exercises of the Southern Medical College was a beautiful drug case from the enterprising house of Parke, Davis & Co., of Detroit, Michigan. The case contained twenty four vials filled with the *normal liquids* and other preparations gotten up in that pure, neat and reliable manner for which this house is noted. The normal liquids from this establishment are becoming exceedingly popular as being of more uniform strength and more reliable quality than the ordinary tinctures or fluid extracts.

The case above mentioned, sent especially to be presented as a premium, is of finest red morocco, elegantly lined and containing the name of the donor in gold gilt. The vials were filled with the following articles :

*Normal Liquids* of—

Aconite root,
Belladona leaves,
Nux vomica,
Gelsemium,
Ipecac,
Tinc. ipecac and opium,
Chlo. anodyne,
C. C. Pills (sugar coated),
Pil. quin. iron and zinc. val.,
Granules of arsenious acid,
Zinc valerian,
Phos. and nux. vomica,
Calomel,
Strychnia,
Podophyllum,
Sulph. quinine,
Sulphate zinc,
Atropine tablets,
Esserine sulphate,
Jalapin,
Pepsin,
Powd. belladonna,
F. Ext. Cascara sagrada.

This beautiful and friendly token from the house of Parke, Davis & Co. to the Southern Medical College is thankfully acknowledged and warmly appreciated.

